# Deep reinforcement learning approaches for global public health strategies for COVID-19 pandemic

**DOI:** 10.1371/journal.pone.0251550

**Published:** 2021-05-13

**Authors:** Gloria Hyunjung Kwak, Lowell Ling, Pan Hui

**Affiliations:** 1 Department of Computer Science and Engineering, The Hong Kong University of Science and Technology, Hong Kong, China; 2 Department of Anaesthesia and Intensive Care, The Chinese University of Hong Kong, Hong Kong, China; 3 Department of Computer Science, University of Helsinki, Helsinki, Finland; Texas A&M University, UNITED STATES

## Abstract

**Background:**

Unprecedented public health measures have been used during this coronavirus 2019 (COVID-19) pandemic to control the spread of SARS-CoV-2 virus. It is a challenge to implement timely and appropriate public health interventions.

**Methods and findings:**

Population and COVID-19 epidemiological data between 21st January 2020 to 15th November 2020 from 216 countries and territories were included with the implemented public health interventions. We used deep reinforcement learning, and the algorithm was trained to enable agents to try to find optimal public health strategies that maximized total reward on controlling the spread of COVID-19. The results suggested by the algorithm were analyzed against the actual timing and intensity of lockdown and travel restrictions. Early implementations of the actual lockdown and travel restriction policies, usually at the time of local index case were associated with less burden of COVID-19. In contrast, our agent suggested to initiate at least minimal intensity of lockdown or travel restriction even before or on the day of the index case in each country and territory. In addition, the agent mostly recommended a combination of lockdown and travel restrictions and higher intensity policies than the policies implemented by governments, but did not always encourage rapid full lockdown and full border closures. The limitation of this study was that it was done with incomplete data due to the emerging COVID-19 epidemic, inconsistent testing and reporting. In addition, our research focuses only on population health benefits by controlling the spread of COVID-19 without balancing the negative impacts of economic and social consequences.

**Interpretation:**

Compared to actual government implementation, our algorithm mostly recommended earlier intensity of lockdown and travel restrictions. Reinforcement learning may be used as a decision support tool for implementation of public health interventions during COVID-19 and future pandemics.

## 1. Introduction

Coronavirus disease 2019 (COVID-19) was first reported by health authorities in Wuhan, China on 31st December 2019 [1]. In mainland China, the number of confirmed infections with severe acute respiratory syndrome coronavirus 2 (SARS-CoV-2), increased to around 75,000 within a month from the first confirmation date of 20th January 2020 [2]. Korea and Italy were the next outbreak countries and currently identified cases have been reported in 216 countries and territories. The massive number of patients infected within a short time period have overwhelmed many countries and territories. The lack of reliable and rapid testing, self-quarantine facilities, personal protective equipment, hospital and critical care capacity and effective treatment have created a health crisis for countries and territories that were not ready. As of 15th April 2021, COVID-19 has caused more than 2,970,000 deaths globally, and this figure is likely a conservative estimate due to under diagnosis. Furthermore, this COVID-19 pandemic and the measures used to control it have resulted in a global crisis affecting across all economic sectors and disruption to mental and social wellbeing [3–5].

Determining the appropriate type and level of public health policy for each country and territory is very challenging. Different countries and territories have their unique population structure and density, economic resources, healthcare systems, governance, and culture. In addition, the index case of COVID-19 and initial spread of the virus for each country and territory is often unknown. Thus, governments were forced to apply policies with incomplete information about the burden of disease as well as the uncertainty about the biological and clinical characteristics of the virus [6]. Decision making is further complicated by the response lag in new infections, hospitalizations, and mortality. In the meantime, studies have investigated the effects of these public health decisions [7–10]. For example, the effect of travel restrictions on domestic and international spread of SARS-CoV-2 was studied with data from 200 countries and territories using the global epidemic and mobility model (GLEAM) [11, 12]. The model showed that 77% reduction in cases imported to other countries due to travel restriction out of Wuhan although it only had modest effect on domestic spread in China. Yet when the efficacy of travel restrictions was assessed at different transmission scenarios, travel ban was only meaningful if combined with a 50% or higher transmission reduction [8]. Early or preemptive lockdown has also been shown to be more effective than delayed response in China [9]. Using simulated data, it was shown that lockdown policy reduces the number of deaths even when only 5% of population is infected [10]. Taken together, it suggests that fast intervention, and simultaneously placing nationwide and worldwide travel bans are effective. However, there is still a lack of effective tools to provide specific decision support for individual countries and territories with different health care systems and burden of COVID-19.

In this work, we propose a data-driven preliminary approach to discover optimal lockdown and travel restriction policies for individual countries and territories with the state-of-the-art deep reinforcement learning (RL) algorithm. Reinforcement learning is one of three basic machine learning fields along with supervised and unsupervised learning. It is based on the concept in the human learning process of what to do in a particular situation: how to map the situation to action. Contrary to the concept of supervised learning to learn the correct action (label) with a description of situation (example), reinforcement learning seeks an action that maximizes accumulated reward received through trial-and-error without being told what to do directly [13–15]. We conducted policy effectiveness studies with deep RL to learn sequential decision making to maximize rewards over time by accelerations and decelerations in the number of confirmed COVID-19 infections, deaths and recovered cases. The timing and intensity of lockdown and travel restriction policies were suggested by the deep RL approaches and compared to actual public health interventions implemented during this COVID-19 pandemic.

## 2. Methods

### 2.1. Data and pre-processing

We included data between 21st January 2020, the first date that the World Health Organization (WHO) reported on COVID-19, to 15th November 2020 from 216 countries and territories. For each country and territory, index case date (date of the first locally confirmed patient), the numbers tested, confirmed infection, recovered and dead were collected from Johns Hopkins coronavirus data repository, Centers for Disease Control and Prevention’s reports and WHO’s case reports [16–18]. We also collected data on timing and intensity of domestic lockdown and international travel restrictions. This included early actions from countries and territories implemented before the first local case of infection was confirmed. Population size, population density, population mid-year (aged 15 to 65 years old), gross domestic product (GDP), geological information (longitude, latitude) and life expectancy from the United Nations database, Wikipedia, and official announcements through the news were used in our algorithm for the country and territory specific population characteristics and healthcare setting [16–22]. After linear interpolation from the index case date in each country and territory, data was compiled with an average value over a 3 day period, to reduce bias from delayed reporting and variable viral testing capacity [23–25]. We chose to use 3 days rather than daily figures since every time stamp required time sensitive information, but at the same time, to reduce bias from delayed reporting and variable viral testing capacity over weekends. Countries and territories were excluded from analysis if they had fewer than 100 cases of COVID-19 by 15th November 2020. After unity-based data normalization for feature scaling, the dataset was divided into a 7:2:1 ratio of training, validation and test sets.

#### 2.1.1. Severity level

The crude death rate due to COVID-19 reported on 15th November 2020, calculated as the number of deaths related to COVID-19 in the total population (per 1,000) was used as an indicator of the country or territory’s overall crisis severity level. The severity group was divided into four levels (low/medium/high/critical level of severity). Countries or territories which did not have any deaths were designated as low severity group. The remaining were evenly divided into 3 groups according to the COVID-19 crude death rate. Each severity groups’ characteristics and burden of COVID-19 is shown in Table 1.

**Table 1:** Table of population, life expectancy and GDP for each severity group.

Severity level	Low	Medium	High	Critical
**Population**	0.2 (0.1–5.2)	27.6 (9.2–52.2)	8.94 (1.87–31.3)	12.2 (1.9–63.8)
**Population (mid-year)**	0.1 (0.0–3.2)	15.4 (5.4–31.8)	5.4 (1.1–18.6)	8.5 (1.6–39.3)
**Population density**	62.0 (20.0–212.5)	79.2 (37.9–270.6)	113.6 (44.0–271.9)	90.5 (46.7–205.9)
**Life expectancy**	73.6 (70.5–78.6)	66.7 (62.6–76.7)	75.4 (72.1–80.0)	78.7 (76.5–82.2)
**GDP (x10**^**3**^**)**	14.7 (4.4–30.6)	3.8 (2.0–18.6)	15.4 (2.0–18.6)	25.2 (14.3–45.3)
**Crude death rate (x10**^**-6**^**)**	0 (0–0)	0.3 (0.0–0.5)	2.1 (0.2–3.6)	9.9 (1.2–20.9)

All values are expressed in median and interquartile range unless specified; Population: Population estimates (millions); Population (mid-year): Population mid-year estimates (millions); Population density (/km^2^); Life expectancy (years); GDP: GDP (PPP) per capita (USD); Crude death rate: the number of deaths related to COVID-19 in the total population reported on 15th November 2020.

### 2.2. Model

The goal of reinforcement learning is to train a decision-making agent to seek to achieve its target (maximizing cumulative rewards) despite uncertainty about its environment [13]. At each time stamp *t*, an agent has a combination of action *a*_*t*_ and state *s*_*t*_ along with reward *r*_*t*_ for each case. By interacting with its environment, at each time stamp *t*, an agent receives state *s*_*t*_ and reward *r*_*t*_ from environment, and then chooses an action *a*_*t*_. Subsequently the action *a*_*t*_ is sent to the environment. The environment moves to the next state *s*_*t*+1_ and finally the agent receives an evaluative feedback *r*_*t*+1_ from the environment. In this way, a reinforcement learning agent tries to maximize cumulative rewards with feedback (reward) received after taking action [13–15]. Reinforcement learning has been widely applied in a variety of fields such as robotics, healthcare, finance and games such as AlphaGo and Atari, and has been successful in achieving human-level performance or even surpassing humans [13–15].

#### 2.2.1. Action and reward

We defined a 3×3 action space for the domestic lockdown and travel restrictions. The lockdown was divided into three levels: no action (Level 0: L0), restricted public social gathering (L1) and nationwide lockdown (L2). Likewise, travel policy covered no action (T0), flight suspension (T1) and full closure of all borders (T2) from each country or territory. Specifically, travel restrictions refer to measures adopted by each country or territory rather than travel bans exerted from others. We focused on how to adjust these interventions on a per-region basis, and also their crucial impact on a country and territory’s severity level (the crude death rate on 15th November 2020).

**Fig 1:**
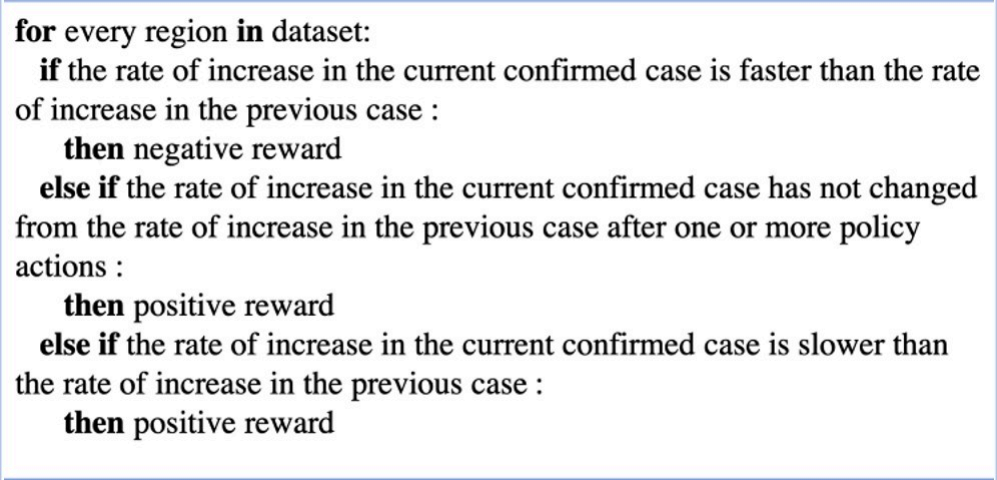
Example for a compensation formula of the confirmed case. If each country and territory had positive or negative acceleration in growth of case, it was rewarded accordingly; For conditions where there was no change in growth rate, positive reward was considered only if there was at least one action or one or more confirmed cases were found to reduce long-term no action impact before the first confirmed case was reported.

Our rewards were designed to punish accelerated increases in cases of infection and death, and to encourage rapid acceleration of recovery cases with a 2:1:1 ratio. The rationale behind this reward system was twofold. First, minimizing increases in new infections is associated with reduced mortality and should receive the highest priority out of the three metrics [26]. Second, there is a significant time lag difference between the onset of new infection and recovery or death, leading to delay in action for governments, which can be considered for action and reward in reinforcement learning [27]. Therefore, we chose to punish increased acceleration of new infections relatively more than increased acceleration of death or compensate for increased acceleration of recovered cases. We assigned positive reward stabilization (no change) or decreasing rates of new infection but negative rewards for increasing rates of new infection cases despite actions. No change in new infection rates was assigned positive rewards because a lack of increase in rates of new cases is often the first sign of stabilization during an outbreak [28]. Fig 1 shows example code for a compensation formula for reward based on confirmed cases. More details on actions and rewards can be found in S1–S5 Figs in S1 File.

#### 2.2.2. Model architecture

In this study, our agent was trained to seek an optimal policy with the Dueling Double Deep Q-Network (D3QN) which is a variant of Deep Q-Network among deep RL algorithms [29–31]. This network was chosen to distinguish the quality of the state *s*_*t*_ (country and territory characteristics and burden of COVID-19) and the chosen action *a*_*t*_ (lockdown and travel policy) at each timestamp *t* without overestimation of high dimension temporal data [14, 30–32]. We used the D3QN primarily for off-policy learning, dueling architecture, overestimation, and replay buffer. Off-policy learning technique was needed to seek an optimal policy from the data generated from other behaviour policies [14]. Double DQN was required to control overestimation [30, 31]. Dueling DQN has two streams which allow us to separately estimate (scalar) the state-value and advantages for each action, so that it can learn the value of states (ex. population density or life expectancy) without considering how each action for each state affects the environment [30, 31]. Finally, replay buffer with Double DQN is more advantageous, since real world data can lead the model in only one way, especially when well-distributed quality data is insufficient [14, 30, 31]. After unity-based data normalization, experiments were conducted up to 100,000 episodes using a mini-batch size of 8, all 13 variables mentioned in Data and pre-processing section and section 6.1 in S1 File, and the final result was selected at the stabilized convergence point with the squared error loss function for the main network and target network. During training, the importance of immediate and future rewards was balanced by maximizing the expected discount return using the discount factor *γ*, and each parameter was updated based on mini-batch and optimal policy was evaluated with samples [31, 33]. For implementation, we used Scikit-learn 0.20.3 library for data pre-processing, and D3QN was adapted from previous research works and optimized for this paper with Keras 2.3.1 and Tensorflow 1.15.0 in Python [31, 32] (More details of the proposed architecture and comparison of architectures can be found in sections 6.3 and 7.1 in S1 File).

#### 2.2.3. Comparison of actual policy to agent decisions

After training the agent to learn the implemented policies and the associated rewards, we derived the suggested initiation date and intensity level of lockdown or travel restriction for each country and territory. Difference in timing of public interventions implemented by governments and those suggested by our agent was assessed by comparing the earliest date of either lockdown or travel restriction. We performed the comparisons twice using two different reference dates. First we used local index case date to reflect each country’s action relative to the start of the local health crisis with COVID-19. Second, we used 31, December 2019 as reference date to assess relative timing of each country or territory’s actions against the start of the global COVID-19 pandemic. In addition, we compared the overall timing and intensity level of these interventions by governments and the deep RL approaches over the duration of the pandemic up to 15th November 2020. We used Susceptible-Infectious-Recovered-Dead (SIRD) model to simulate scenarios to evaluate how reinforcement learning may help reduce burden of COVID-19 [34].

## 3. Results

### 3.1. Timing of policies

**Fig 2:**
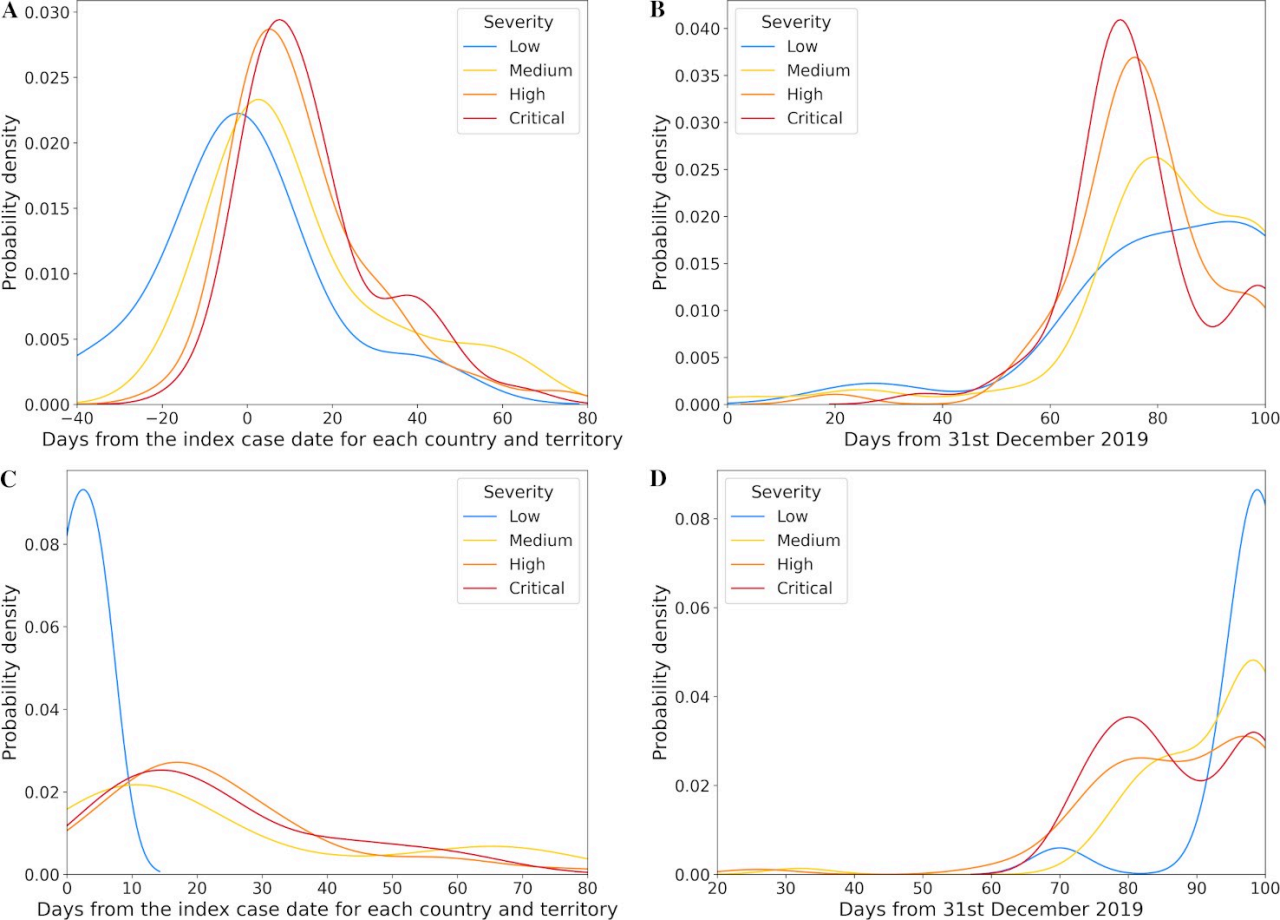
Distribution (kernel density estimate) of lockdown policy date. A. Any lockdown policy date from the index case date for each country and territory and B. from 31st December 2019; C. Full lockdown policy date from the index case date for each country and territory and D. from 31st December 2019.

**Fig 3:**
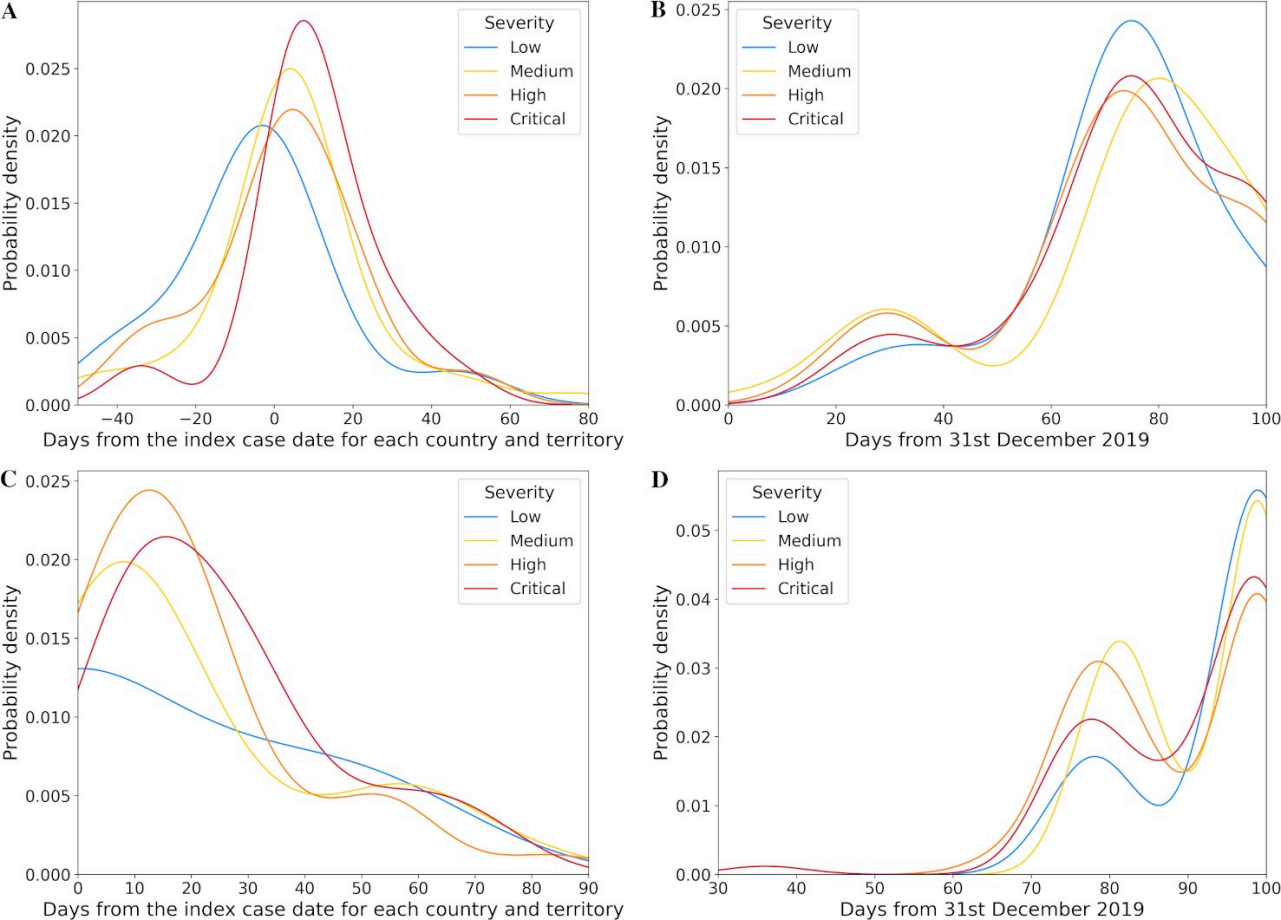
Distribution (kernel density estimate) of travel restriction policy date. A. Any travel restriction policy date from the index case date for each country and territory and B. from 31st December 2019; C. Full travel restriction policy date from the index case date for each country and territory and D. from 31st December 2019.

The actual timing of lockdown and travel restriction policies for each severity level relative to 31st December 2019 and the index case date for each country and territory are shown in Figs 2 and 3. Even prior to their local index case, some countries and territories applied initial lockdown measures in their community or some form of travel restrictions (Figs 2A and 3A). Some of them reported their first COVID-19 patient before mid-March. Full lockdowns (L2) and closure of all borders (T2) were always only applied after index case date in each country or territory. Overall, early implementation of any or full lockdown and travel restriction policies were associated with progressively lower levels of crisis severity. These relationships were only apparent when considering timing relative to local index case date in each country or territory (Figs 2A and 2C and 3A and 3C) and were not present when using 31st December 2019 as the reference date (Figs 2B and 2D and 3B and 3D).

**Fig 4:**
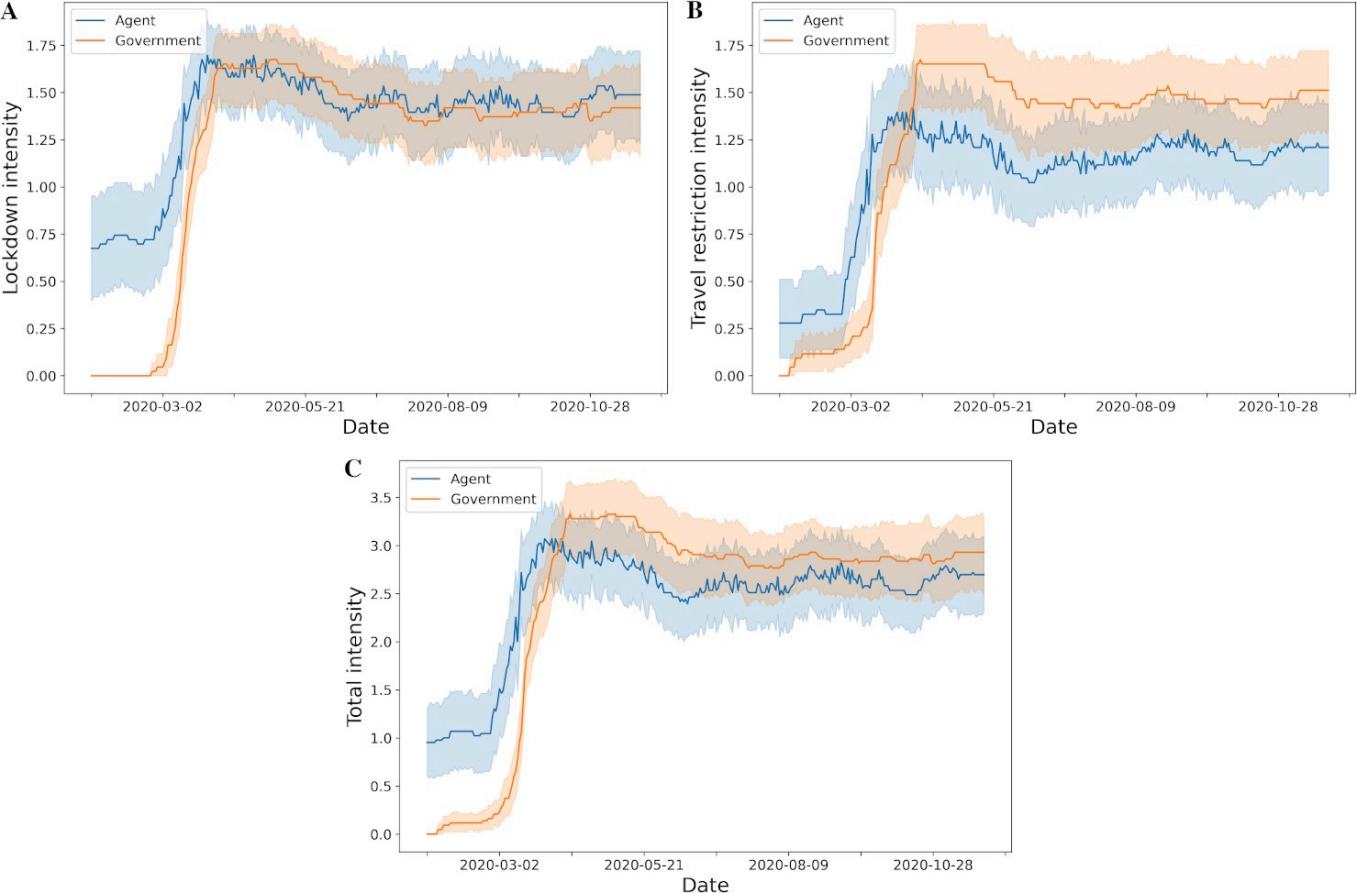
Lockdown and travel restriction policy intensity from government and agent over time. A. Lockdown policy; B. Travel restriction policy; C. Total policy which is the sum of lockdown and travel restriction policies (the mean and 95% confidence interval).

**Fig 5:**
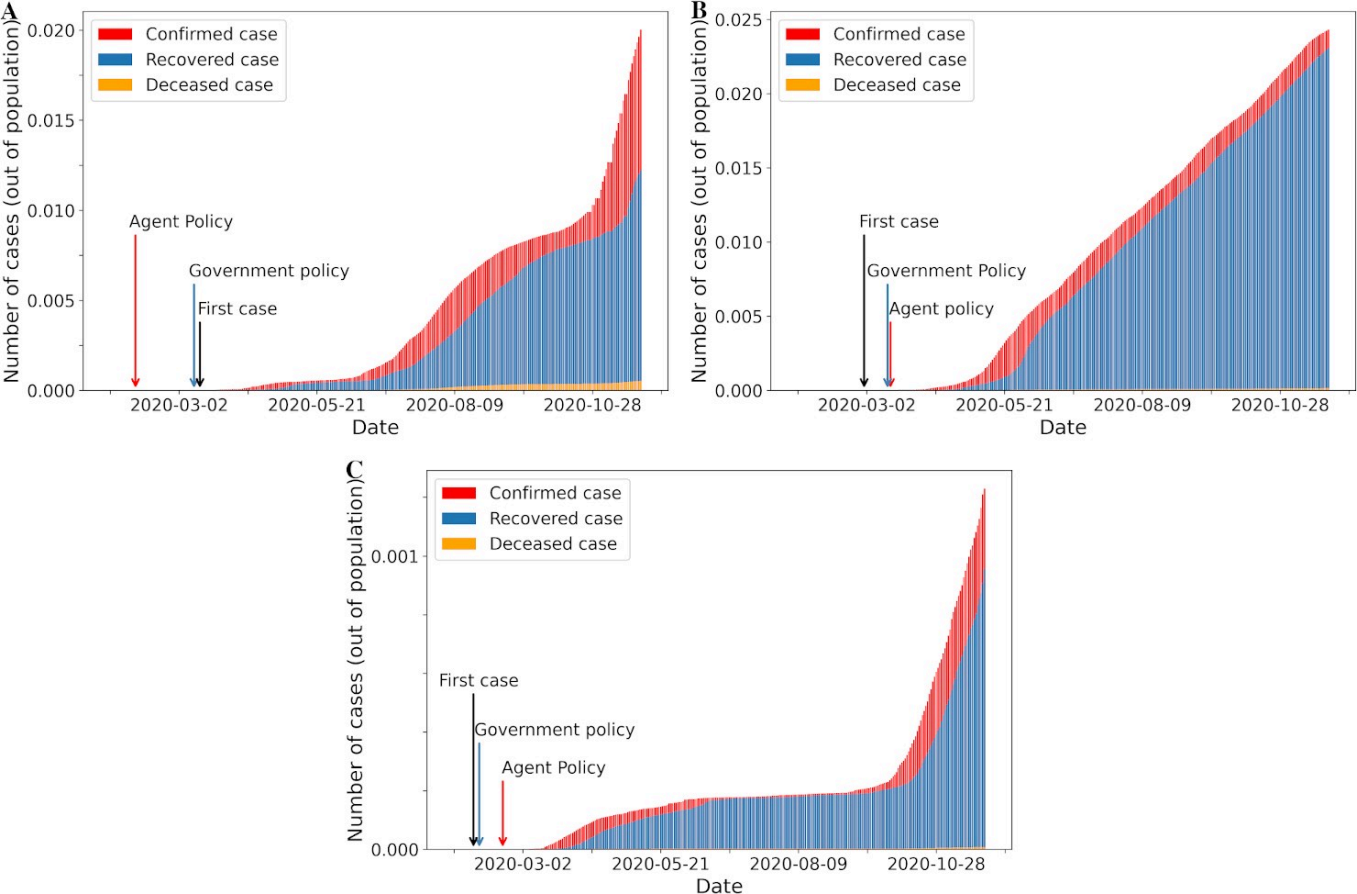
Number of cases over time according to difference between agent and government policy. A. First agent policy before government policy; B. First government and agent policy after index case; C. First government policy before agent policy.

The overall intensities level of lockdown and travel restriction policies implemented by governments and suggested by our agent through the reinforcement learning over the course of the pandemic are shown in Fig 4. In general, the agent proposed lockdown or travel restriction policy at level one earlier than when it was actually implemented by governments (Fig 4C). Our agent suggested to initiate at least minimal intensity of lockdown or travel restriction even before or on the day of the index case in each country and territory (S6 Fig in S1 File). For examples, in some countries and territories, the agent recommended that the first policy at any level should be implemented in late January or early February, even if the index case date was in mid- or late March (Fig 5A). Interestingly, this coincides with the travel ban from Wuhan, China on 23rd January 2020 [8]. In addition, proposed action timing from the agent did not deviate from the actual implementation dates for some countries and territories (Fig 5B). In contrast, for some countries and territories, the agent suggested to delay policy implementation whereas governments took early action even though the number of cases did not grow exponentially (Fig 5C).

### 3.2. Intensity of restrictions

In general the intensity of both lockdown and travel restriction polices suggested by our agent were higher than government policies until April 2020 (Fig 4). Of note, during the early days of the pandemic, the 95% confidence intervals were wide for our agent whilst government had narrow intervals. This corresponded to the time period when many governments have not initiated any policies. This difference in variance of policy intensity between government and agent was reduced over the course of the pandemic. Overall, the agent opted for an earlier and shorter maximum lockdown and travel restriction (L2, T2) than governments.

### 3.3 Significance of policy differences

**Fig 6:**
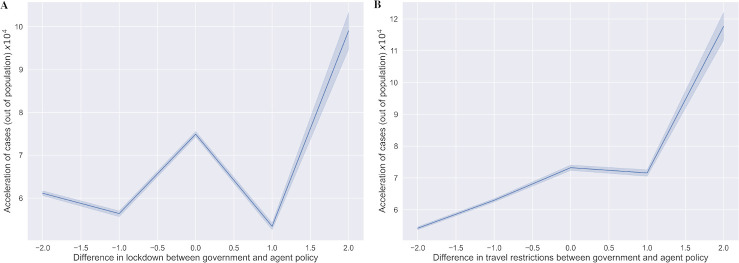
Relationship between acceleration in COVID-19 burden and the difference in policy levels between the government and agent. Between 21st January 2020 to 15th November 2020, the difference between the government and agent at any time (recommended policy level by agent minus the given policy level by government) was calculated. We plotted the association between the difference and the COVID-19 burden, defined as acceleration of new infected cases, acceleration of death cases, and deceleration of recovery cases (with a 2:1:1 ratio) in the total population per 1,000,000 progressed. Overall, acceleration of COVID-19 burden at any time occurred when government policies were less intense than suggested by agent; A. lockdown policy; B. travel restriction policy.

For policy validation, we used an evaluation technique to estimate how the differences between the government and agent policies relate to accelerated infection, death and recovery cases [32, 33]. The total acceleration was calculated and derived in relation to the difference at the policy level. In general, earlier agent lockdowns when compared to government lockdown policy was related to a more rapid acceleration in cases (Fig 6A and 6B). In contrast, earlier government closure of border compared to agent was not necessarily associated with slower acceleration of cases. This may be a reflection of local transmission having greater influence on burden of disease when compared to imported cases.

### 3.4 Policy comparison using different learning period data

**Fig 7:**
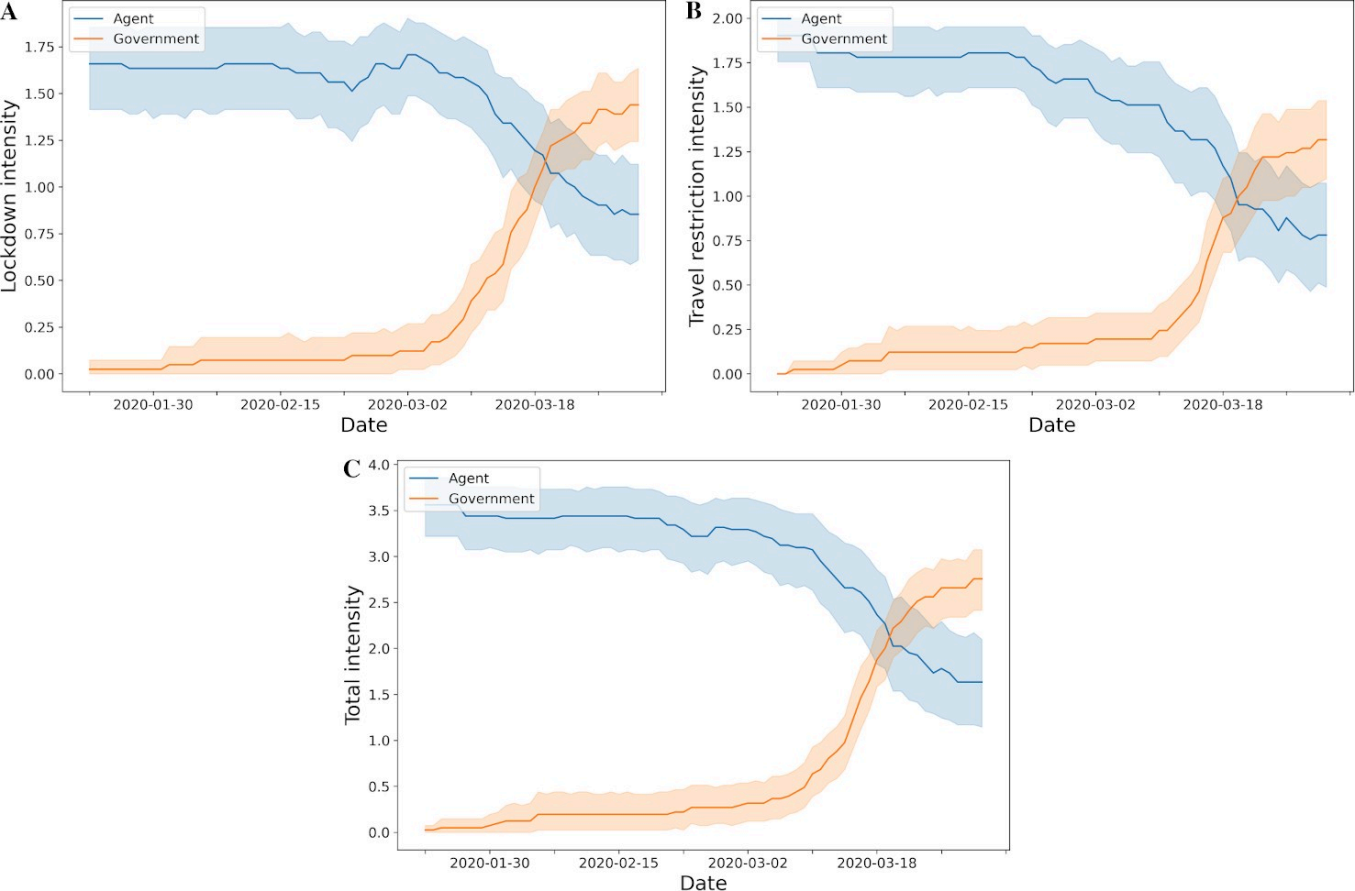
Lockdown and travel restriction policy intensity from government and agent over time (first three months). A. Lockdown policy; B. Travel restriction policy; C. Total policy which is the sum of lockdown and travel restriction policies (the mean and 95% confidence interval).

**Fig 8:**
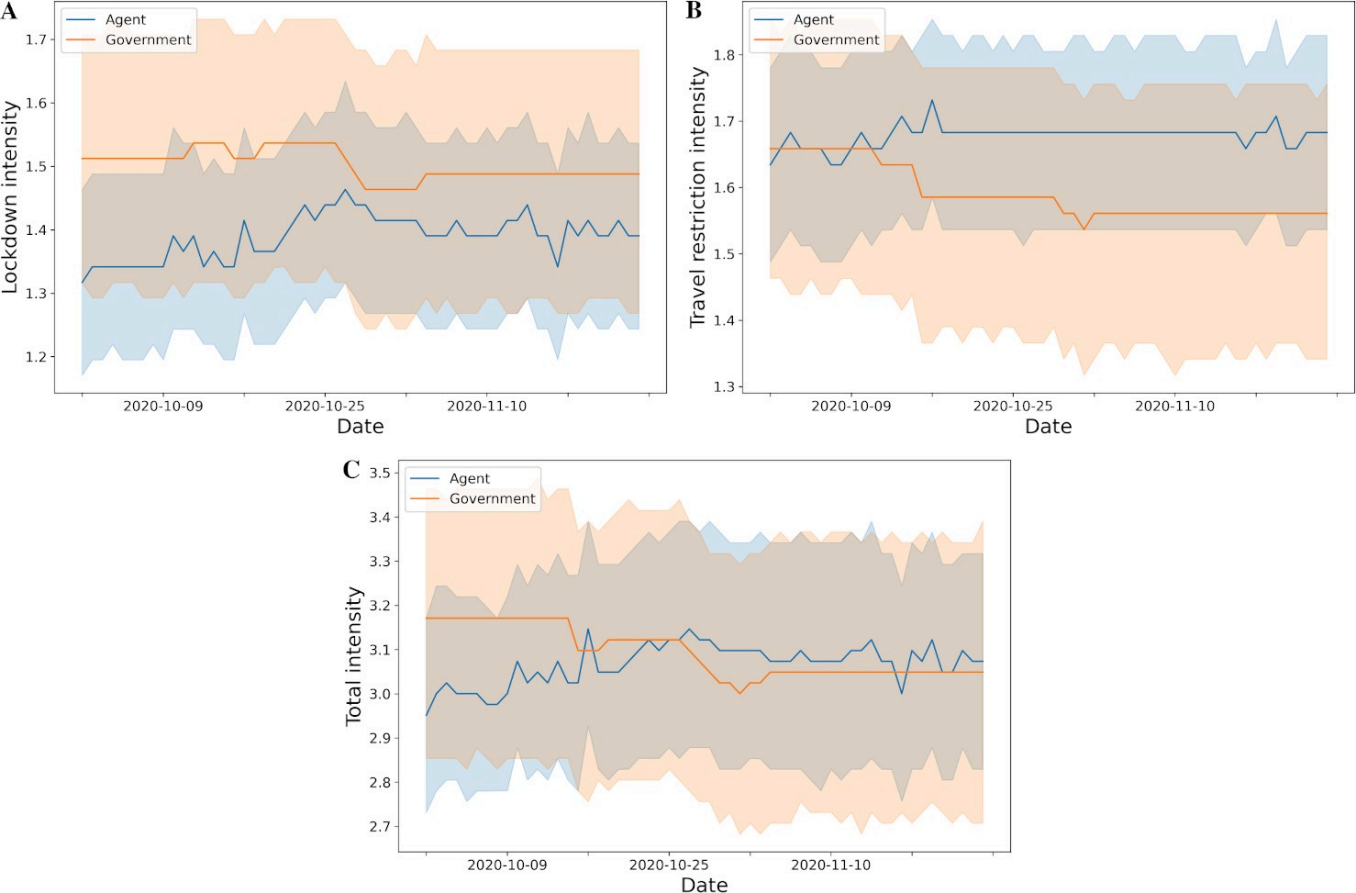
Lockdown and travel restriction policy intensity from government and agent over time (most recent 3 months). A. Lockdown policy; B. Travel restriction policy; C. Total policy which is the sum of lockdown and travel restriction policies (the mean and 95% confidence interval).

We trained the proposed network and analyzed the results based on three different periods of pandemic data (first 3 months, full period, most recent 3 months). In Fig 7, when we trained only using data from the first three months, the agent initially proposed to maintain high regulation of local and international policies, but from mid-March the intensity of the proposed policies was reduced. Policies at the minimum level were proposed at the end of March. Meanwhile, the policies proposed in the most recent three months have reached some degree of agreement with the policies proposed by the governments in Fig 8. Specifically, the level of proposed lockdown policy was slightly lighter than government policy, and travel restriction was slightly higher than government policy.

## 4. Discussion

In this study we used deep RL on country and territory population data and serial local COVID-19 epidemiological data to develop an algorithm to train an agent to determine the optimal timing and intensity of lockdown and travel restriction for individual countries and territories. We performed timing analysis of policy implementation for each crisis severity and deep RL with continuous state space and rewards to find the suitable action for each state at a particular point in time. When compared to actual government implementation during the COVID-19 pandemic, our algorithm mostly recommended earlier intensity of lockdown and travel ban.

During an emerging pandemic, it is a challenge to implement timely and appropriate public health interventions with limited data. Early on during the pandemic, SARS-CoV-2 transmission kinetics were unknown. Furthermore, the efficacy and principal of social distancing, lockdowns, and international travel restrictions have been questioned [35]. The results in this paper are consistent with previous studies which suggests lockdown and travel restrictions are effective in reducing the transmission of SARS-CoV-2 [8, 9]. We have shown that adoption of the proposed policies using reinforcement learning may help reduce burden of COVID-19 through scenario simulator results with SIRD model (S7 Fig in S1 File) [34].

During the early phase of the pandemic, our agent suggested earlier and higher intensity of lockdown to control the pandemic (Fig 4A). However, over time the agent agreed with the government policies. Furthermore, it agreed with governments to lower lockdown policy intensities in the later stage of the pandemic. This is important because adopting policies early to reduce burden of COVID-19 has to be balanced against the economic, social and health concerns [3, 36–41]. Even with punishment such as fines and imprisonment for contravening public health policy, it is difficult to sustain lockdowns and border closures over long periods. In addition, for some countries and regions, the agent suggested to warn the citizens with an early low intensity lockdown such as public gathering limits or encouraging online e-learning, but not always recommending a full lockdown overall. Similarly, the agent recommended at least level 1 travel restrictions (T1) early when compared to government implantation (Fig 4B). Interestingly, governments implemented and maintained higher intensity of travel restrictions compared to agent’s recommendations that it may be relaxed over time. The algorithm and results of this study suggests that high intensity lockdown and travel restrictions do not need to be applied to all countries and territories over sustained periods. However, whilst this is encouraging, this may be because some countries and territories have low floating populations or other defense strategies from other countries or territories have an effect on these countries and territories.

To further analyze the results from the proposed algorithm, the results were compared and evaluated by using pandemic data from three different training periods (first 3 months, full period, most recent 3 months) in Section 3.4 with Figs 4, 7 and 8. Fig 7 showed that when the algorithm training was derived from data obtained during the first three months, agents initially proposed to maintain high regulation policies, and then reduce the intensity from mid-March. In Fig 8, using the pandemic data from the most recent three months, agents have reached some degree of agreement with the decisions made by the governments, similar to the proposed policies trained over the entire period (Fig 4), but there were contradictions regarding travel restrictions. The travel restriction policy learned over the entire period was slightly lower than that of the government during the second half of the study period. But when only data from the last three months is considered (no action was taken initially), the travel restriction policy proposed by agent was slightly stronger than the government (Fig 8). This is related to the fact that in the case of Fig 4, the lockdown and travel restriction policies learned over the entire period were given a faster and higher level in the early days of the pandemic, and the policies were mostly maintained thereafter. In conclusion, these results (Figs 4 and 8) and the results of the first three months (Fig 7) all suggest that initiating a policy in the first place can reduce many kinds of losses. It also shows that countries and territories can start with similarly strong policies even now and return to a minimum level in a short time.

The contributions of this study include the use of deep RL to evaluate the effects of public interventions on spread of COVID-19 using real world epidemiological and population data. This approach utilizes reward based on targets to “flatten the curve” to learn the optimal timing and intensity of policies. Optimizing the particular timing and intensity of policies using reinforcement learning approaches have been previously studied [32, 42, 43]. Specifically, reinforcement learning has been used to simulate effect of lockdown in COVID-19 [10, 14, 15]. Instead of using supervised learning which depends on reliably labelled data, we used deep reinforcement learning to learn sequential decision-making with successive steps. Although it is not the most recent ‘state-of-the-art’, the reinforcement learning architecture provides an agent that learns the values of states, and balances the importance of immediate and future rewards by maximizing the expected discount return using the discount factor *γ* [31]. The algorithm also performs updates on each parameter based on mini-batch and evaluates the recommended policy using samples [31, 33]. Despite neighbouring countries and territories reporting cases, many governments chose to enact lockdown or travel bans only after the first local confirmed case. Instead, our agent suggested the policy timing even before the first local case by considering what has happened in other countries and territories, near and afar. The differences in policies are likely due to significant political and economical consequences of travel bans and lockdowns versus doubts on efficacy of these policies on controlling local transmission during the early phase of the pandemic. Conversely, our off-policy learning based algorithm is impartial, objective, and proactive, trying to find an out-of-the-box optimal approach to control the pandemic for both the near and far future. Hence, reinforcement learning is particularly useful in a developing global pandemic, when the resolution is not clear. Countries which do not have reported cases can learn from other countries’ timing and intensity of public interventions, and the efficacy of these actions. As shown in Fig 6, reinforcement learning found the optimal policies according to the temporal and population characteristics unique to each country and territory that minimized the burden of COVID-19. The result is an individualized recommendation on timing and intensity of lockdown and travel restriction for each country and territory based on global burden of disease.

The limitation of the current paper is that it was carried out with imperfect data due to the emerging COVID-19 pandemic. Inconsistent reporting of confirmed cases underestimates local burden of COVID-19, whilst increased testing capacity over time will cause an apparent rise in confirmed cases even though community spread may be stagnant. It is expected that more solid results will be obtained as we learn more about the transmission kinetics of the virus, the clinical characteristics of COVID-19, and have consistent testing and higher fidelity population data. In addition, in some countries there was additional provincial data collected, but country-level data had to be used to maintain consistency and avoid problems caused by incomplete data. We were also unable to analyze the impact on individual travel restrictions on other countries and territories. With more detailed data on travel restrictions it may be possible to separate instances where travel bans between countries and territories have influenced each other. Only the official lockdown policies were available, and the official policy may differ from those practiced in the community. Also, policy evaluation in this paper requires credibility in clinical decision making for the proposed policy decision, which is difficult [33, 44, 45]. With an emphasis on exploring policy interpretation possibilities and application directions, evaluation methods were adopted in previous studies [32, 33, 46, 47]. Implementation of the newly proposed interpretable reinforcement learning and further simulation studies on reinforcement learning to examine parameters (ex. reward function) that balance economic and population health impacts should be considered as research directions [48, 49]. Lastly, we were not able to strike a balance between policy decisions for public health and negative impacts such as economic consequences as these remain to be determined. This was because we were unable to separate the economic and social costs related to COVID-19 pandemic from the consequences of public policies used to control the virus spread. Our research focuses only on the population health benefits of controlling the spread of COVID-19. Nevertheless, we have shown that reinforcement learning may be used to learn the effect of public health interventions.

## 5. Conclusion

In this study, we used deep RL to learn efficacy of lockdown and travel restrictions in controlling the COVID-19 crisis. Using local population and COVID-19 epidemiological data, we showed that the algorithm can be trained to have an agent to find the optimal strategy in specific countries and territories to maximize the expected value of total rewards over time. Compared to actual government policy implementation, the agent mainly proposed to have earlier lockdown and travel restrictions to reduce the burden of COVID-19.

## Supporting information

S1 File (DOCX)

## References

[1] Wu JT, Leung K, Leung GM. Nowcasting and forecasting the potential domestic and international spread of the 2019-nCoV outbreak originating in Wuhan, China: a modelling study. Lancet [Internet]. 2020 2 29;395(10225):689–97. Available from: 10.1016/S0140-6736(20)30260-9 32014114 PMC7159271

[2] The Center for Systems Science and Engineering, Johns Hopkins. Coronavirus COVID-19 Global Cases. https://coronavirus.jhu.edu

[3] Nicola M, Alsafi Z, Sohrabi C, Kerwan A, Al-Jabir A, Iosifidis C, et al. The Socio-Economic Implications of the Coronavirus and COVID-19 Pandemic: A Review. Int J Surg. 2020 10.1016/j.ijsu.2020.04.018 32305533 PMC7162753

[4] Serafini G, Parmigiani B, Amerio A, Aguglia A, Sher L, Amore M. The psychological impact of COVID-19 on the mental health in the general population. QJM An Int J Med. 2020;113(8):531–7 10.1093/qjmed/hcaa201 32569360 PMC7337855

[5] Poudel K, Subedi P. Impact of COVID-19 pandemic on socioeconomic and mental health aspects in Nepal. Int J Soc Psychiatry. 2020 10.1177/0020764020942247 32650687 PMC7443960

[6] Cascella M, Rajnik M, Cuomo A, Dulebohn SC, Di Napoli R. Features, evaluation and treatment coronavirus (COVID-19). In: Statpearls [internet]. StatPearls Publishing; 2020 32150360

[7] Kucharski AJ, Russell TW, Diamond C, Liu Y, Edmunds J, Funk S, et al. Early dynamics of transmission and control of COVID-19: a mathematical modelling study. Lancet Infect Dis [Internet]. 2020 4 22; Available from: 10.1016/S1473-3099(20)30144-4 32171059 PMC7158569

[8] Chinazzi M, Davis JT, Ajelli M, Gioannini C, Litvinova M, Merler S, et al. The effect of travel restrictions on the spread of the 2019 novel coronavirus (COVID-19) outbreak. Science. 2020;368(6489):395–400 10.1126/science.aba9757 32144116 PMC7164386

[9] Tian H, Liu Y, Li Y, Wu C-H, Chen B, Kraemer MUG, et al. An investigation of transmission control measures during the first 50 days of the COVID-19 epidemic in China. Science. 2020;368(6491):638–42 10.1126/science.abb6105 32234804 PMC7164389

[10] Khadilkar H, Ganu T, Seetharam DP. Optimising Lockdown Policies for Epidemic Control using Reinforcement Learning. arXiv Prepr arXiv200314093. 2020 10.1007/s41403-020-00129-3 PMC7311597 38624387

[11] Balcan D, Colizza V, Gonçalves B, Hu H, Ramasco JJ, Vespignani A. Multiscale mobility networks and the spatial spreading of infectious diseases. Proc Natl Acad Sci USA [Internet]. 2009 12 22;106(51):21484–9. Available from: 10.1073/pnas.0906910106 20018697 PMC2793313

[12] Balcan D, Gonçalves B, Hu H, Ramasco JJ, Colizza V, Vespignani A. Modeling the spatial spread of infectious diseases: The GLobal Epidemic and Mobility computational model. J Comput Sci [Internet]. 2010;1(3):132–45. Available from: 10.1016/j.jocs.2010.07.002 21415939 PMC3056392

[13] Sutton RS, Barto AG. Reinforcement learning: An introduction. 2011

[14] Li Y. Reinforcement learning applications. arXiv Prepr arXiv190806973. 2019

[15] Yu C, Liu J, Nemati S. Reinforcement learning in healthcare: a survey. arXiv Prepr arXiv190808796. 2019

[16] Dong E, Du H, Gardner L. An interactive web-based dashboard to track COVID-19 in real time. Lancet Infect Dis. 2020 5 1;20(5):533–4. Available from: 10.1016/S1473-3099(20)30120-1 32087114 PMC7159018

[17] World Health Organization. Novel Coronavirus–Situation Report. 2020. https://www.who.int/emergencies/diseases/novel-coronavirus-2019

[18] Korea Centers for Disease Control and Prevention. The updates on novel Coronavirus infection in Korea. 2020. https://www.cdc.go.kr

[19] United Nations. United Nations Data Retrieval System. 2020. https://data.un.org

[20] Wikipdedia. 2019–20 coronavirus pandemic. 2020. https://en.wikipedia.org/wiki/2019%E2%80%9320_coronavirus_pandemic

[21] Max R, Hannah R, steban O, Joe H. Coronavirus Pandemic (COVID-19). 2020. https://ourworldindata.org/coronavirus

[22] Hale T, Webster S, Petherick A, Phillips T and Kira B. Oxford COVID-19 Government Response Tracker. Blavatnik School of Government. 2020 10.1038/s41562-021-01079-8 33686204

[23] Liu J, Zhou J, Yao J, Zhang X, Li L, Xu X, et al. Impact of meteorological factors on the COVID-19 transmission: A multi-city study in China. Sci Total Environ [Internet]. 2020;726:138513. Available from: 10.1016/j.scitotenv.2020.138513 32304942 PMC7194892

[24] Ediriweera DS, de Silva NR, Malavige GN, de Silva HJ. An epidemiological model to aid decision-making for COVID-19 control in Sri Lanka. PLoS One [Internet]. 2020;15(8):e0238340. Available from: 10.1371/journal.pone.0238340 32853295 PMC7451571

[25] Bukhari Q, Jameel Y, Massaro JM, D’Agostino RB, Khan S. Periodic Oscillations in Daily Reported Infections and Deaths for Coronavirus Disease 2019. JAMA Netw open. 2020;3(8):e2017521 10.1001/jamanetworkopen.2020.17521 32804210 PMC7431996

[26] Kenyon C. Flattening-the-curve associated with reduced COVID-19 case fatality rates- an ecological analysis of 65 countries. J Infect. 2020;81(1):e98–99. Available from: 10.1016/j.jinf.2020.04.007 32305488 PMC7162747

[27] Manuel B, Richard K, Sarah T, Hans HH, Andreas FW, Richard AN. 2019-Novel Coronavirus (2019-nCoV): estimating the case fatality rate–a word of caution. Swiss Med Wkly. 2020;150:w20203. Available from: 10.4414/smw.2020.20203 32031234

[28] Tang Y, Wang S. Mathematic modeling of COVID-19 in the United States. Emerg Microbes Infect. 2020;9(1):827–9 10.1080/22221751.2020.1760146 32338150 PMC7241447

[29] Mnih V, Kavukcuoglu K, Silver D, Graves A, Antonoglou I, Wierstra D, et al. Playing atari with deep reinforcement learning. arXiv Prepr arXiv13125602. 2013

[30] Van Hasselt H, Guez A, Silver D. Deep reinforcement learning with double q-learning. In: Proceedings of the AAAI Conference on Artificial Intelligence. 2016

[31] Wang Z, Schaul T, Hessel M, Hasselt H, Lanctot M, Freitas N. Dueling network architectures for deep reinforcement learning. In: International conference on machine learning. PMLR; 2016;2016:1995–2003

[32] Raghu A, Komorowski M, Ahmed I, Celi L, Szolovits P, Ghassemi M. Deep reinforcement learning for sepsis treatment. arXiv Prepr arXiv171109602. 2017

[33] Jiang N, Li L. Doubly robust off-policy value evaluation for reinforcement learning. 33rd Int Conf Mach Learn ICML. 2016;2:1022–35

[34] Kermack WO, McKendrick AG. A Contribution to the Mathematical Theory of Epidemics. Proc R Soc. 1927;115:200–721

[35] Xiao Y, Torok ME. Taking the right measures to control COVID-19. Lancet Infect Dis. 2020 10.1016/S1473-3099(20)30152-3 32145766 PMC7128408

[36] Holmes EA, O’Connor RC, Perry VH, Tracey I, Wessely S, Arseneault L, et al. Multidisciplinary research priorities for the COVID-19 pandemic: a call for action for mental health science. The Lancet Psychiatry. 2020 10.1016/S2215-0366(20)30168-1 PMC7159850 32304649

[37] Cetron M, Landwirth J. Public health and ethical considerations in planning for quarantine. Yale J Biol Med. 2005;78(5):329 17132339 PMC2259156

[38] Wilder-Smith A, Freedman DO. Isolation, quarantine, social distancing and community containment: pivotal role for old-style public health measures in the novel coronavirus (2019-nCoV) outbreak. J Travel Med. 2020;27(2):taaa020 10.1093/jtm/taaa020 32052841 PMC7107565

[39] Sohrabi C, Alsafi Z, O’Neill N, Khan M, Kerwan A, Al-Jabir A, et al. World Health Organization declares global emergency: A review of the 2019 novel coronavirus (COVID-19). Int J Surg. 2020 10.1016/j.ijsu.2020.02.034 PMC7105032 32112977

[40] Reger MA, Stanley IH, Joiner TE. Suicide Mortality and Coronavirus Disease 2019—A Perfect Storm? JAMA psychiatry. 2020 10.1001/jamapsychiatry.2020.1060 32275300

[41] Qiu J, Shen B, Zhao M, Wang Z, Xie B, Xu Y. A nationwide survey of psychological distress among Chinese people in the COVID-19 epidemic: implications and policy recommendations. Gen psychiatry. 2020;33(2) 10.1136/gpsych-2020-100213 32215365 PMC7061893

[42] Watts J, Khojandi A, Vasudevan R, Ramdhani R. Optimizing Individualized Treatment Planning for Parkinson’s Disease Using Deep Reinforcement Learning. 2020;5406–9 10.1109/EMBC44109.2020.9175311 33019203

[43] Zhao Q, Xu C, Jin S. Traffic Signal Timing via Parallel Reinforcement Learning. Smart Innov Syst Technol. 2019;149(3):113–23

[44] Murphy SA, van der Laan MJ, Robins JM, CPPRG. Marginal Mean Models for Dynamic Regimes. J Am Stat Assoc [Internet]. 2001;96(456):1410–23. Available from: 10.1198/016214501753382327 20019887 PMC2794446

[45] Hirano K, Imbens GW, Ridder G. Efficient estimation of average treatment effects using the estimated propensity score. Econometrica. 2003;71(4):1161–89

[46] Yu C, Liu J, Zhao H. Inverse reinforcement learning for intelligent mechanical ventilation and sedative dosing in intensive care units. BMC Med Inform Decis Mak. 2019;19 10.1186/s12911-019-0737-8 30961594 PMC6454602

[47] Gottesman O, Johansson F, Meier J, Dent J, Lee D, Srinivasan S, et al. Evaluating Reinforcement Learning Algorithms in Observational Health Settings. 2018;1–16. Available from: http://arxiv.org/abs/1805.12298

[48] D’Orazio M, Bernardini G, Quagliarini E. How to restart? An agent-based simulation model towards the definition of strategies for COVID-19 “second phase” in public buildings. 2020;1–21. Available from: http://arxiv.org/abs/2004.12927

[49] Srinivasan S, Doshi-Velez F. Interpretable Batch IRL to Extract Clinician Goals in ICU Hypotension Management. AMIA Jt Summits Transl Sci proceedings AMIA Jt Summits Transl Sci [Internet]. 2020;2020:636–45. Available from: http://www.ncbi.nlm.nih.gov/pubmed/32477686 32477686 PMC7233064

