# Supplementary material for: Deep reinforcement learning approaches for global public health strategies for COVID-19 pandemic
Source: PLoS One. 2021 May 13;16(5):e0251550. doi: 10.1371/journal.pone.0251550 (PMC8118301; doi:10.1371/journal.pone.0251550)
Supplement: S1 File — (DOCX) [file pone.0251550.s001.docx]

S1 File for

Deep reinforcement learning approaches for global public health strategies for COVID-19 pandemic

Gloria Hyunjung, Kwak, Lowell Ling, Pan Hui.

Correspondence to: hkwak@cse.ust.hk (G.H.K.), lowell.ling@cuhk.edu.hk (L.L.)

**This PDF file includes:**

Section 6. Model

Section 7. Simulator Model

Figs. S1 to S8

**6. Model**

Q-learning is a model-free RL, where an agent learns which action is good to execute at a particular state. Over the years, Deep Q-Network have addressed shortcomings such as high dimensionality problems with a deep convolutional neural network. Double Q-Network solved $Q$-value overestimation, whereas Dueling Q-Network distinguished the quality of the current state (a value function $V$) and the chosen action (a state-dependent action advantage $A$) [31]. Since Dueling Double Deep Q-Network (D3QN) comprehensively characterizes architectures, it was used in this project along with priority experience replay that was proposed together in the paper [29]. Experience replay is to focus on the fact that agents tend to forget certain experiences in the past, to save and replay experiences $(s,a,r,s^{'})$ to effectively use the experience and improve training convergence performance. Focusing on the highest loss experience is called priority experience replay.

**6.1. Features**

We used the number of confirmed infections, recovery and deaths, acceleration of each case, growth population size, population density, year-round population, gross domestic product (GDP), geological information (longitude, latitude), and life expectancy as features in our algorithm. At each of these time steps, a 13 × 1 feature vector was used as the state $s_{t}$ for each country or territory.

**6.2. Action and Reward**

For the action space, we defined a 3$\times$3 action space with discretizing each of the domestic lockdown and travel restrictions from level 0 to 2. The combination of these two actions results in 9 possible action choices for every 3-day interval.

A reward function was designed to punish accelerated increases in number of new infections and deaths and encourage accelerated increases in recovered cases using a 2:1:1 ratio. This function and the ratio were used as indicators of the local burden of COVID-19 and the ability of each country or territory to respond to the health crises. For example, if increases in cases of infection increased more rapidly for each time step, we gave a negative reward, such as $-c_{0}-c_{1}\times$ (changes in the rate of increase in infection confirmed cases). Likewise, if the number of patients recovering increased faster than before, there were a positive reward, such as $0.5c_{0}+0.5c_{1}\times$ (changes in the rate of increase in recovery cases). Basically, the basis of this compensation policy is to give delicate compensation depending on whether the action is performed and how much change, even with the same rate of change, in addition to the standard compensation based on a 2:1:1 ratio. We opted for the design of the reward function for all time steps as follows:

$r_{t}=r_{t}^{crc}+0.5\times r_{t}^{crd}+0.5\times r_{t}^{crr}$
($crc,crd,and crr :confirmed infection,death,and recovery cases)$

$$r_{t}^{i}=\begin{matrix} \left\{ \begin{aligned} -c_{0}-c_{1}\times(s_{t+1}^{i}-s_{t}^{i}), &if (s_{t+1}^{i}>s_{t}^{i}) and (s_{t}^{i}>0) \\ -{0.5\times c}_{0}+c_{1}\times(s_{t+1}^{i}-s_{t}^{i}), &if {(s}_{t+1}^{i}=s_{t}^{i}) and (s_{t+1}^{i}\neq0) \\ and ((a_{t+1}^{ld}>0) or (a_{t+1}^{tr}>0)) \\ c_{0}-c_{1}\times(s_{t+1}^{i}-s_{t}^{i}), &if {(s}_{t+1}^{i}<s_{t}^{i}) \\ {-c}_{1}\times(s_{t+1}^{i}-s_{t}^{i}), &otherwise \end{aligned} \right. \\ for i in crc and crd \end{matrix}$$

$$r_{t}^{i}=\begin{matrix} \left\{ \begin{aligned} c_{0}+c_{1}\times(s_{t+1}^{i}-s_{t}^{i}), &if (s_{t+1}^{i}>s_{t}^{i}) and (s_{t}^{i}>0) \\ 0.5\times c_{0}+c_{1}\times(s_{t+1}^{i}-s_{t}^{i}), &if {(s}_{t+1}^{i}=s_{t}^{i}) and (s_{t+1}^{i}\neq0) \\ and ((a_{t+1}^{ld}>0) or (a_{t+1}^{tr}>0)) \\ -c_{0}+c_{1}\times(s_{t+1}^{i}-s_{t}^{i}), &if {(s}_{t+1}^{i}<s_{t}^{i}) \\ -c_{1}\times(s_{t+1}^{i}-s_{t}^{i}), &otherwise \end{aligned} \right. \\ for i in crr \end{matrix}$$

$$r_{t}=reward at timestamp t$$

$$s_{t}=state (acceleration of cases) at timestamp t$$

$$c_{0},c_{1}=constant value \left( ex.100, 50 respectively \right)$$

$$a_{t}^{ld}, a_{t}^{tr}=lockdown and travel restriction action for each at timestamp t$$

**6.3. Model architecture**

In this study, our agent was trained to seek an optimal policy with the Dueling Double Deep Q-Network (D3QN) which is a variant of Deep Q-Network among deep RL algorithms. The architecture and training flow are illustrated in Figs S1 and S2. We experimented the architecture with 52 and 104 (out of 52, 104, and 130) size of units for two hidden layers MLP, using $Q$-value threshold $\left( ex.\pm c_{0} \right)$, batch size 8 (out of 4, 8, and 16) for batch normalization, and probability epsilon $\epsilon$ 0.01. The parameters were selected based on the convergence of the error rates. For the policy, an $\epsilon$-greedy policy was used to choose a random action with probability $\epsilon$ or an action according to the optimal $Q$ function with probability $1-\epsilon$. We ensured the number of cases so that each severity level (the crude death rate) and action level was proportionately present in all training, validation and testing sets. The architecture and parameters were chosen to encourage avoiding saturation and overfitting (overestimation) and to make the most of the case change rates. We used the loss function using the weights ($\theta, \theta^{'}$, respectively) for the main network and the target network as follows:

$$L\left( \theta\right)\mathbb{=E}\left[ \left( Q_{target}-Q\left( s,a;\theta\right) \right)^{2} \right]+\lambda\cdot\left| Q\left( s,a;\theta\right)-Q_{threshold} \right|$$

$$Q_{target}=r+\gamma Q\left( s^{'},\underset{a^{'}}{\mathrm{argmax}} Q\left( s^{'},a^{'};\theta\right);\theta^{'} \right)$$

Experience replay is to save and replay experiences $\left( s,a,r,s^{'} \right)$ to use it and improve training convergence performance effectively. Focusing on the highest loss experience is called priority experience replay, and it is used in this study. For the off-policy value evaluation, Doubly Robust Off-policy Value Evaluation was used [30,32]. We studied how the policy given by a government and an agent were different at every timestamp, and the tendency of rewards (cases’ change rates) based on that. The unbiased estimator of $\mathcal{V}^{\mathcal{H}}$ is based on the recursion formula as below where $\mathcal{H}$ is the history of the country or territory, $\mathcal{N}$ is the number of countries and territories, $\rho_{t}$ is importance ratio per timestamp at time $t$, and $\pi$ is the probabilities of taking policy for action under given state representation:

$$\mathcal{V}^{\mathcal{H+}\boldsymbol{1-}\boldsymbol{t}}\boldsymbol{:=}\hat{\mathcal{V}}\boldsymbol{(}\boldsymbol{s}_{\boldsymbol{t}}\boldsymbol{)+}\boldsymbol{\rho}_{\boldsymbol{t}}\left( \boldsymbol{r}_{\boldsymbol{t}}\boldsymbol{+}\boldsymbol{\gamma}\mathcal{V}^{\mathcal{H-}\boldsymbol{t}}\boldsymbol{-}\hat{\boldsymbol{Q}}\boldsymbol{(}\boldsymbol{s}_{\boldsymbol{t}}\boldsymbol{,}\boldsymbol{a}_{\boldsymbol{t}}\boldsymbol{)} \right)$$

$$\boldsymbol{\rho}_{\boldsymbol{t}}\boldsymbol{:=}\frac{\boldsymbol{\pi}_{\boldsymbol{1}}\left( \boldsymbol{a}_{\boldsymbol{t}} | \boldsymbol{s}_{\boldsymbol{t}} \right)}{\boldsymbol{\pi}_{\boldsymbol{0}}\left( \boldsymbol{a}_{\boldsymbol{t}} | \boldsymbol{s}_{\boldsymbol{t}} \right)}$$

**7. Results**

**7.1. Model comparison**

In this study, different reinforcement learning methods (DQN, DDQN: Double DQN, D3QN) were used to learn the public health policies and validate the performance, feasibility, and generalizability of a trained D3QN model, as well as the effectiveness of dueling network and double Q-learning techniques. A comparative experiment was conducted, and the action $Q$-value changes are shown in S8 Fig.

We demonstrated how much D3QN avoids overestimation and helps in terms of policy management, by evaluating the robustness and generalization ability of the trained agents. Specifically, the proposed D3QN algorithm was compared to DQN and DDQN using the same parameters under the same scenario described in 10,000 episodes. Figure S8 shows the averaged predicted $Q$-values during the training processes of the DQN, DDQN, and D3QN agents. These curves were obtained by training in 10,000 episodes under the same conditions, and we reflected the overestimations in DQN by the $Q$-values. Comparing the red learning curve of D3QN with the orange curve of DDQN and blue curve of DQN in Fig S8, we found that D3QN algorithm can more efficiently and consistently reduce the averaged predicted $Q$-values of current policy. This represented the fact that D3QN algorithm reduced overestimations more efficiently than other algorithms, and it can be seen that our D3QN-based model is effective in solving public health policy problems.

**8. Simulator Model**

To see the influence of the selected behavior in the algorithm, an additional Susceptible-Infectious-Recovered-Dead (SIRD) model was run [33]. The SIRD model is a composite of the following differential equations.

$$\frac{d\mathcal{S}}{d\mathcal{t}}=-\frac{\beta\mathcal{IS}}{\mathcal{N}}$$

$$\frac{d\mathcal{I}}{d\mathcal{t}}=\frac{\beta\mathcal{IS}}{\mathcal{N}}-\gamma\mathcal{I-}\mu\mathcal{I}$$

$$\frac{d\mathcal{R}}{d\mathcal{t}}=\gamma\mathcal{I}$$

$$\frac{d\mathcal{D}}{d\mathcal{t}}=\mu\mathcal{I}$$

where $\beta,\gamma, \mu$ are the rates of infection, recovery, and mortality, respectively.

To minimize errors due to differences between the effects we expect and how much of a policy affected by complex variables (ex. economic and social consequences) actually affects reality, we used the worst-case scenario possible. All the possible policy impacts are either the same or better than the worst-case scenario policy effect.

$$all possible policy impacts\geq worst-case scenario policy effect$$

Accordingly, in the period in which at least one policy of any level in a country or territory was applied, the maximum infection rate and mortality rate were selected, but the minimum recovery rate was selected for $\beta,\gamma,$and $\mu$. Figure S7A and S7B show that adoption of the proposed actions using RL can help reduce the burden of COVID-19 through scenario simulator results, compared to the timing and intensity of given government policies.


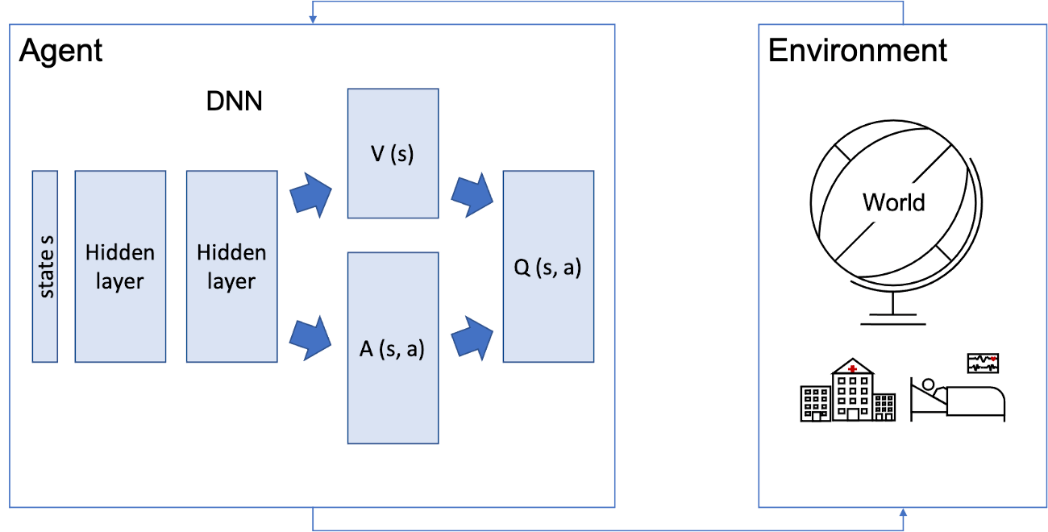


S1 Fig.

**The architecture of our workflow based on D3QN.**


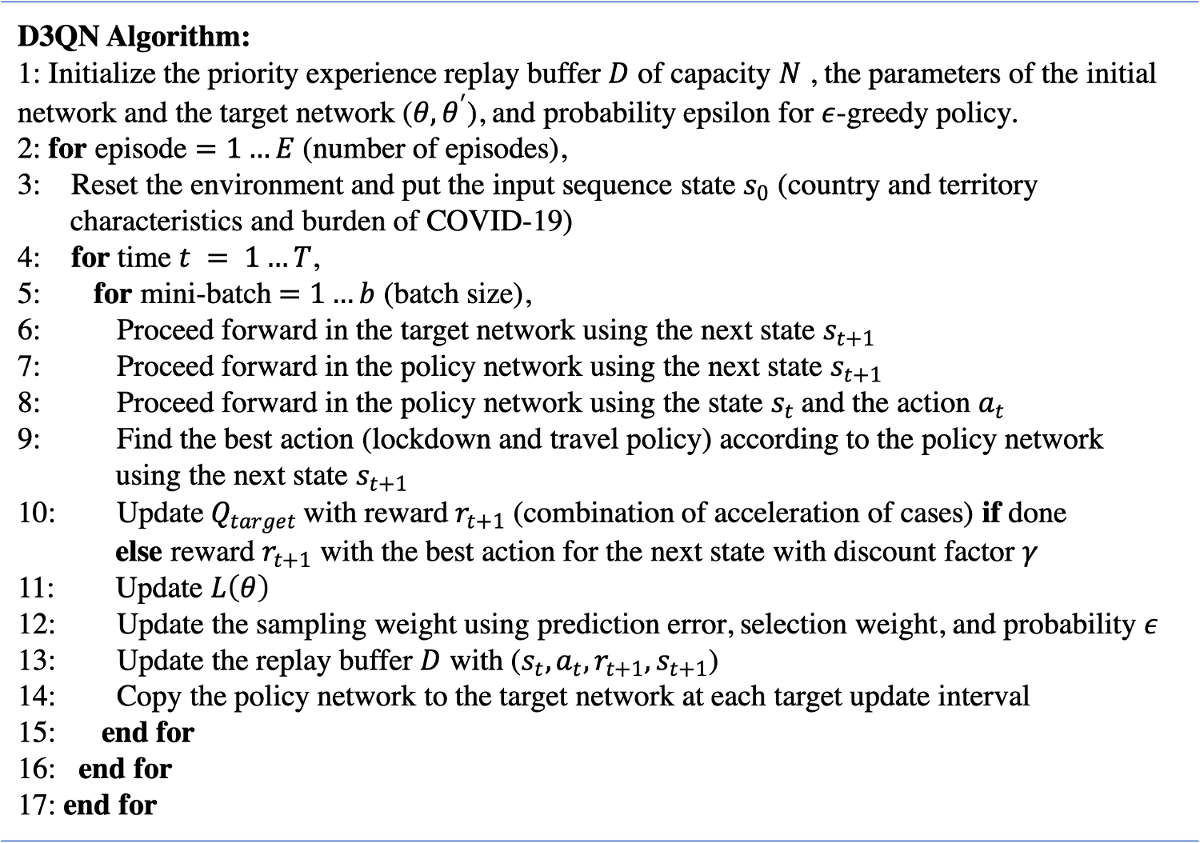


S2 Fig.

**Pseudo code for D3QN implementation.**


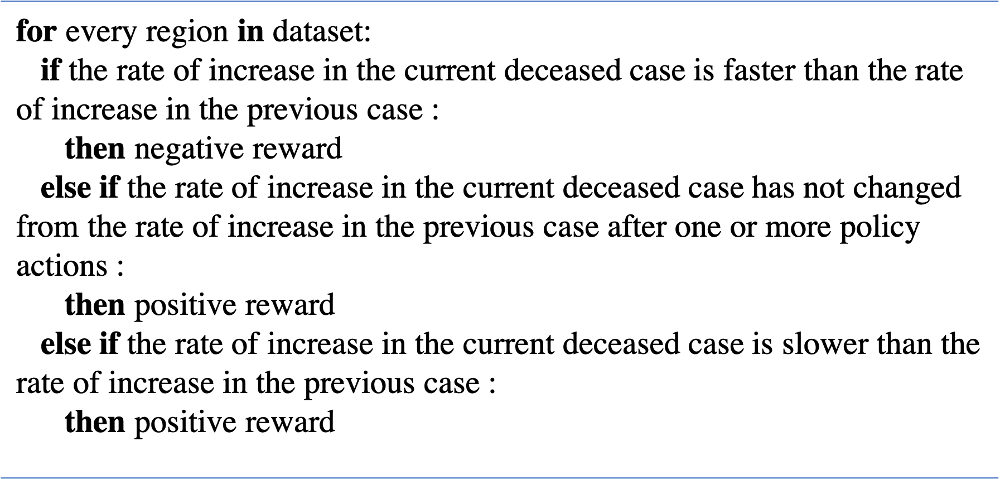


S3 Fig.

Pseudo code for a compensation formula of the deceased case; If each country and territory had positive or negative acceleration in growth of case, it was rewarded accordingly; For conditions where there was no change in growth rate, positive reward was considered only if there was at least one action or one or more deceased cases were found to reduce long-term no action impact before the first deceased case was reported.


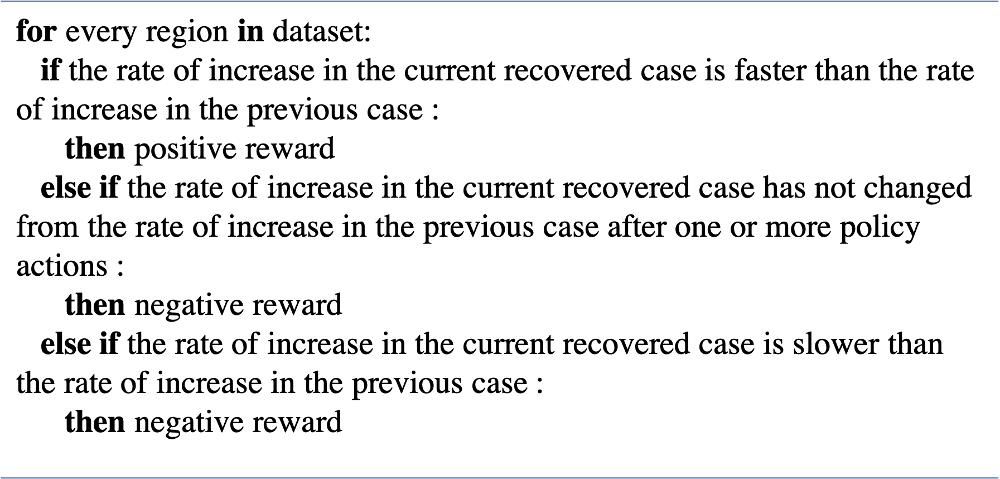


S4 Fig.

Pseudo code for a compensation formula of the recovered case; If each country and territory had positive or negative acceleration in growth of case, it was rewarded accordingly; For conditions where there was no change in growth rate, negative reward was considered only if there was at least one action or one or more recovered cases were found to reduce long-term no action impact before the first recovered case was reported.


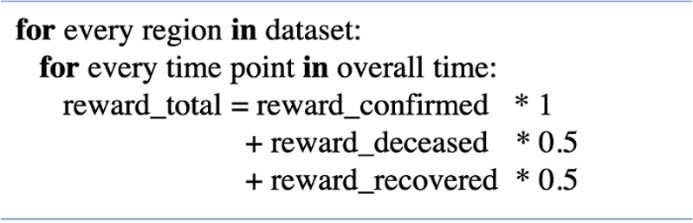


S5 Fig.

**Pseudocode for a total compensation formula covering the confirmed, deceased and recovered cases.**


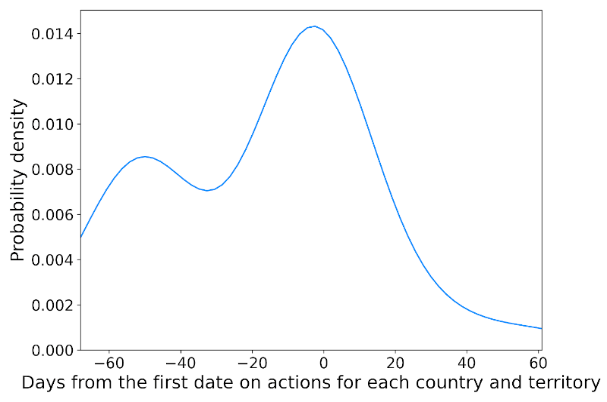


S6 Fig.

**Distribution (kernel density estimate) of suggestions for action before or after any first policy date.**

**
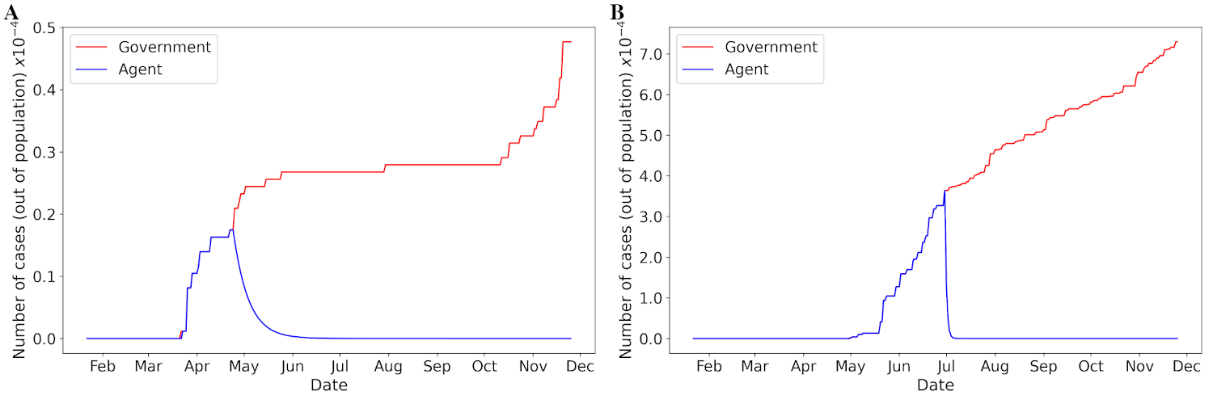
**

S7 Fig.

**Examples of simulated scenarios (A, B) on how number of patients with confirmed infections would change if agent policies were adopted.**


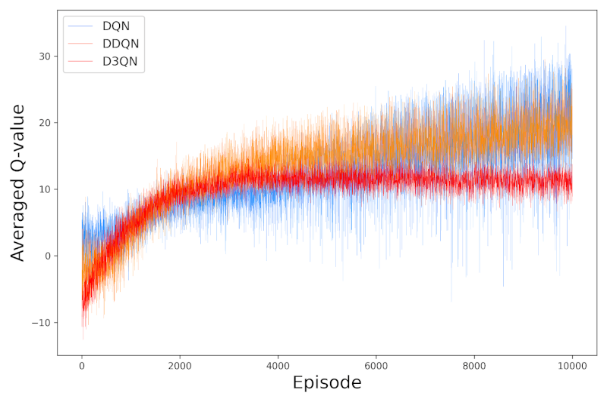


S8 Fig.

Averaged estimated Q-values in training process.
